# *Epimeria liui* sp. nov., a new calcified amphipod (Amphipoda, Amphilochidea, Epimeriidae) from a seamount of the Caroline Plate, NW Pacific

**DOI:** 10.3897/zookeys.922.49141

**Published:** 2020-03-25

**Authors:** Yanrong Wang, Chaodong Zhu, Zhongli Sha, Xianqiu Ren

**Affiliations:** 1 Key Laboratory of Zoological Systematics and Evolution, Institute of Zoology, Chinese Academy of Sciences, Beijing 100101, China Institute of Zoology, Chinese Academy of Sciences Beijing China; 2 Institute of Oceanology, Chinese Academy of Sciences, Qingdao 266071, China Institute of Oceanology, Chinese Academy of Sciences Qingdao China; 3 Laboratory for Marine Biology and Biotechnology, Qingdao National Laboratory for Marine Science and Technology, Qingdao, China University of Chinese Academy of Sciences Beijing China; 4 Center for Ocean Mega-Science, Chinese Academy of Sciences, Qingdao 266071, China Qingdao National Laboratory for Marine Science and Technology Qingdao China; 5 College of Biological Sciences, University of Chinese Academy of Sciences, Beijing 100049, China Center for Ocean Mega-Science, Chinese Academy of Sciences Qingdao China

**Keywords:** Amphipoda, Caroline Plate, deep sea, *
Epimeria
*, new species, seamount, systematics

## Abstract

A calcified individual of *Epimeria* Costa, 1851 collected from an unnamed seamount of the Caroline Plate, NW Pacific, is recognized as new to science herein. This increases the number of known *Epimeria* species of the North Pacific to nine. *Epimeria liui***sp. nov.** differs from its similar congeners by having a rostrum hardly reaching to the end margin of first peduncular article of antenna 1, the presence of large pyriform eyes, the size-increasing mid-dorsal teeth starting from pereonite 6 to pleonite 2, the projection on coxa 5 not extending to epimeral plate 1, and by having a nearly quadrate telson notched medially. To facilitate identification the new species is included in a key to Pacific species of *Epimeria*.

## Introduction

The genus *Epimeria* Costa in Hope, 1851 currently contains nine subgenera and 85 described species (WoRMS 2019). This almost cosmopolitan genus is particularly diverse in the Southern Ocean (59 species), and has been recorded from the intertidal down to 5695 m depth (Stephensen 1947; Shimomura and Tomikawa 2016; d’Udekem d’Acoz and Verheye 2017). When the Chinese research vessel KEXUE surveyed the biodiversity of seamounts on the Caroline Plate, NW Pacific during 2019, one individual referable to *Epimeria* was collected. The specimen exhibits some distinctive characters differentiating it from other described *Epimeria* species, so it is identified as new to science herein. This new species is described, and morphologically compared to other very similar species are presented, and a key to all Pacific *Epimeria* species is also provided.

## Material and methods

The present material was collected by ROV FAXIAN, during expeditions to seamounts on the Caroline Plate by the Institute of Oceanology, Chinese Academy of Sciences (IOCAS) during June to July 2019. The specimen is deposited in the Marine Biological Museum, Chinese Academy of Sciences, Qingdao, China. The individual was examined and dissected with a dissecting microscope (ZEISS Discovery V20). Line drawings were completed using the software Adobe Photoshop CS6 with a graphics tablet. Length measurement was made along the outline of the animal, beginning from the rostrum to the posterior margin of telson.

## Systematics

**Order Amphipoda Latreille, 1816**

**Suborder Amphilochidea Boeck, 1871**

**Superfamily Iphimedioidea Boeck, 1871**

**Family Epimeriidae Boeck, 1871**

### Genus Epimeria

Taxon classificationAnimaliaAmphipodaEpimeriidae

Costa in Hope, 1851

0267D1D3-A7AF-52EF-BD6E-C8D31B86A71D

#### Diagnosis

(from d’Udekem d’Acoz and Verheye 2017). Body smooth or covered with teeth or processes, but not sword-like or forming large longitudinal carinae. Head with developed ventral lobe; rostrum usually well developed; eyes usually present, bulging. Antenna 1 peduncular articles short, with accessory flagellum. Upper lip entire or symmetrically notched. Mandible with incisor and molar present; lacinia mobilis present on both mandibles. Lower lip without inner lobes. Maxilla 1 with 2-articulate palp. Coxae 1–4 progressively longer, coxae 1–3 narrow, coxa 4 five-sided; coxae 5–6 with or without tooth or process projecting laterally. Gnathopods weak; gnathopod 2 longer than gnathopod 1. Pereiopod 6 > pereiopod 5 > pereiopod 7; basis of that with longitudinal carina on both sides. Coxal gill from gnathopod 2 to pereiopod 7. Oostegite large, from gnathopod 2 to pereiopod 6. Uropods well developed. Urosomite 1 always with a rounded or tooth-like process. Telson incised or cleft, rarely emarginate or entire.

### Epimeria liui sp. nov.

Taxon classificationAnimaliaAmphipodaEpimeriidae

F0127C21-29A2-5F47-B94C-FD9EA648B989

http://zoobank.org/419AB34B-9A78-4AAE-9D4B-D18F716134E9

Figs 1 , 2 , 3 , 4

#### Material examined.

**Holotype.** Ovigerous ♀ (17.8 mm) (MBM 286613), dissected, unnamed seamount on Caroline Plate, NW Pacific, M6089, St. FX-Dive 218, 10°07'N, 140°14'E, depth 813–1242 m, 6 June 2019, collected by team of ROV FAXIAN.

#### Diagnosis.

Rostrum hardly reaching to distal margin of first peduncular article of antenna 1; eyes present, pigmented, pyriform. Maxilliped palp article 4 with more than two teeth in internal margin. Coxa 5 with posterodistal corner produced. Pereonites 6, 7 and pleonites 1, 2 with size-increasing mid-dorsal teeth, the one on pereonite 6 blunt and small.

**Figure 1.**
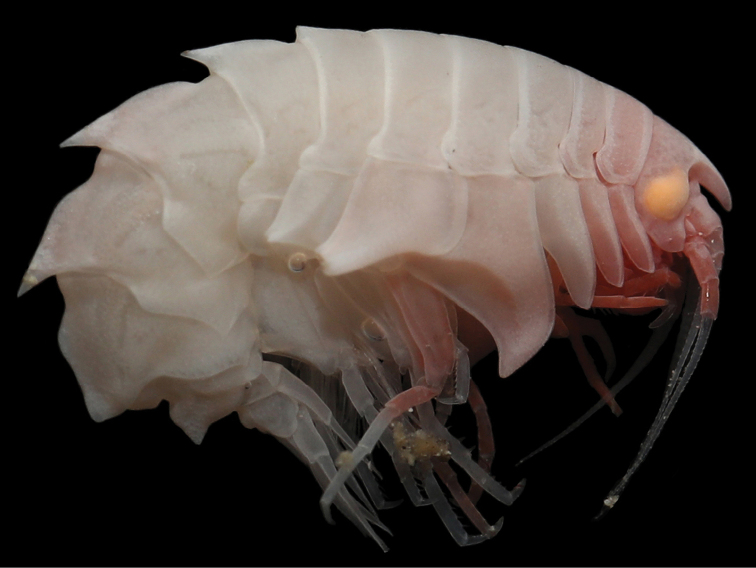
*Epimeria liui* sp. nov., female holotype (17.8 mm) (MBM 286613), photographed immediately after capture by Shao-qing Wang.

#### Description.

Body calcified. ***Head.*** Rostrum nearly as long as head, not reaching to distal margin of first peduncular article of antenna 1; anterior cephalic margin with a small lobe medially, lateral cephalic slightly produced; eyes bulging on head, pigmented, pyriform. Antenna 1 with peduncular article 1 about twice as long as article 2, 3 times as long as article 3, without distal tooth; accessory flagellum scale-like, hardly reaching to half-length of first flagellar article; primary flagellum with 26 articles, sparsely setose. Antenna 2 nearly as long as antenna 1, peduncular article 4 slightly longer than article 5; flagellum with 29 articles.

***Mouthparts.*** Mandible with incisor and lacinia mobilis strongly dentate; molar triturative; palp article 3 densely setose medially, with two long setae distally. Maxilla 1 with inner plate subtriangular, obliquely convex inner margin with 10 stout plumose setae; outer plate distal margin oblique, with 11 lobate robust setae; palp exceeding outer plate; palp 2-articulate, article 2 with 3 robust setae and 5 long setae distally, inner margin bearing row of dense setae. Maxilla 2 with long, slender setae distally on lateral and medial plates. Maxilliped with outer plate broadly rounded distally, bearing short setae, hardly reaching to distal margin of palp article 3; inner plate with row of short setae medially and anteriorly; palp medial margin strongly setose, article 3 with groups of long setae reaching distal end of dactylus, dactylus with serrate medial margin.

**Figure 2.**
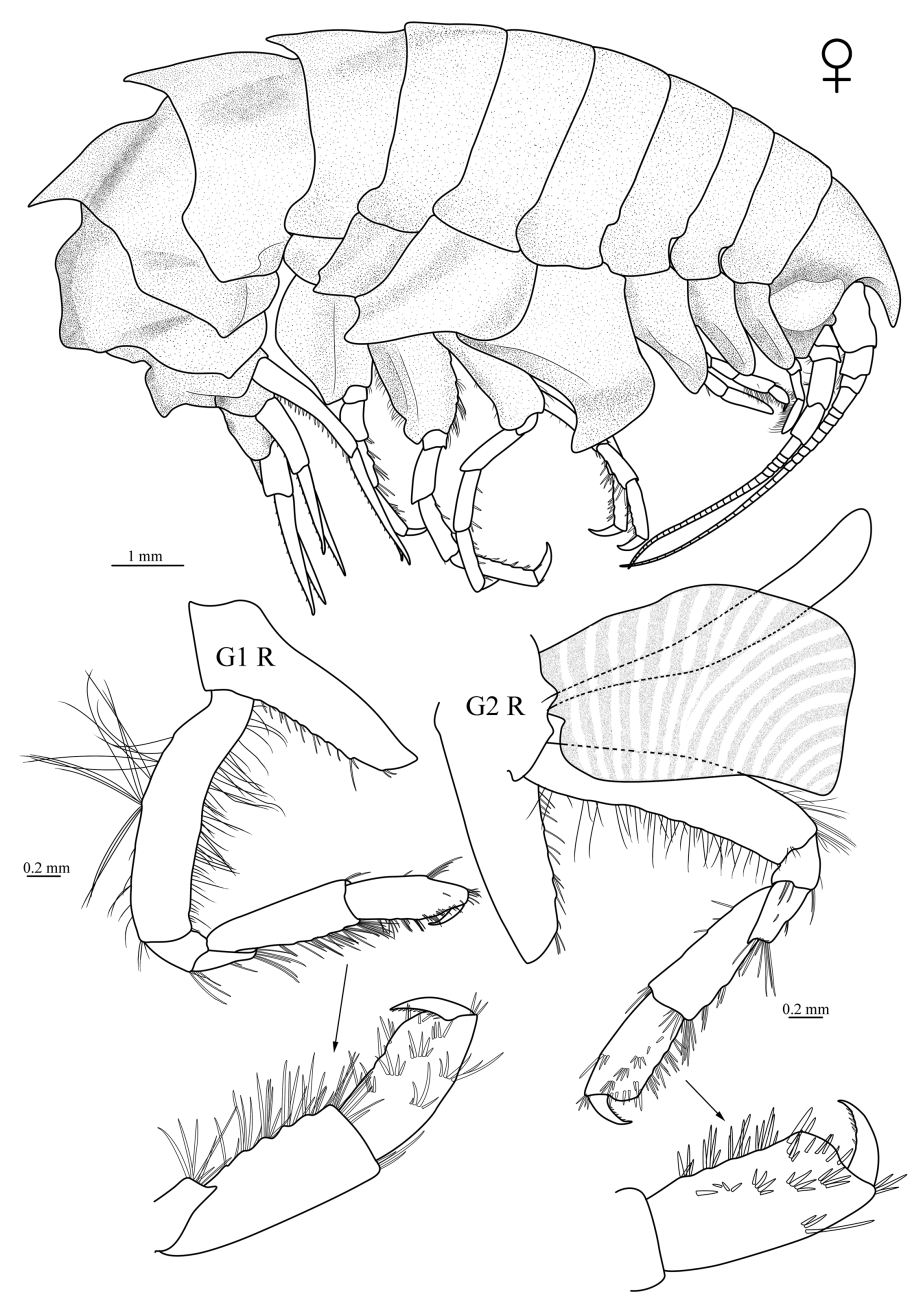
*Epimeria liui* sp. nov., female holotype (17.8 mm) (MBM 286613), G1 R, right gnathopod 1; G2 R, right gnathopod 2.

***Pereonites.*** Pereonites 1–7 lacking lateral projection; pereonite 1 subequal in length to head (excluding rostrum), pereonite 2 shorter than pereonite 1; pereonites 1–5 lacking mid-dorsal tooth; pereonite 6 with slight blunt mid-dorsal protrusion; pereonite 7 with acute triangular mid-dorsal tooth.

***Pleosome.*** Pleonites 1 and 2 with size-increasing, acute triangular mid-dorsal tooth, and inconspicuous posterolateral protrusions; dorsal margin of pleonite 3 sinuous. Epimeral plates 1–3 with posteroventral angle produced into small subacute tooth.

***Urosome.*** Urosomite 1 with blunt triangular mid-dorsal tooth; urosomite 2 shortest; urosomite 3 dorsal margin slightly sinuous.

***Pereopods.*** Gnathopod 1 coxa long and slender, posterior margin bearing row of small robust setae; basis linear, both margins with numerous slender setae; merus nearly as long as ischium, anterior margin very short, distal margin oblique, posterodistal angle acute, setose; carpus linear, longer than propodus, posterior margin strong setose, anterior margin bearing group of setae distally; propodus slightly expanded distally, posterior margin and palm with robust setae, faces bearing groups of robust setae; dactylus slender, slightly curved, posterior margin minutely serrated. Gnathopod 2 coxa wider and longer than coxa 1, posterior margin bearing row of small robust setae; basis linear, ischium and merus similar to that of gnathopod 1; carpus linear, posterior margin setose; propodus and dactylus of similar appearance to gnathopod 1. Pereopod 3 coxa wider and longer than coxa 2, posterior margin bearing small robust setae and blunt protrusion on proximal half; basis linear, both margins setose; merus longer than carpus, margins bearing small setae; carpus shorter than propodus, margins setose; propodus with posterior margin bearing robust setae; dactylus stout, curved, without setae. Pereopod 4 coxa longer than coxa 3, anterior margin nearly straight, ventral tooth slightly curved, apically subacute and oriented backwards, lateral carina without tooth, not projecting laterally, carina very distant from margin of coxa at its deepest point; basis to dactylus as for pereopod 3. Pereopod 5 coxa subrectangular, posterodistal corner produced, drawn out to pointed wing in dorsal view; basis wider than that of pereopod 4, posterodistal corner rounded, setose; ischium bearing posterodistal lobe; merus nearly as long as carpus, posterior margin produced, anterior margin bearing small setae; carpus shorter than propodus, with anterior margin bearing robust setae; propodus with anterior margin setose; dactylus stout, curved. Pereopod 6 coxa bearing carinate, lateral tooth forming a small triangular wing in dorsal view; basis wider in pereopod 5, bearing carina, setose; ischium to dactylus as for pereopod 5. Pereopod 7 coxa subrectangular; basis larger than that of pereopod 6, expanded mid-posteriorly; ischium to dactylus similar to that of pereopods 5 and 6.

**Figure 3.**
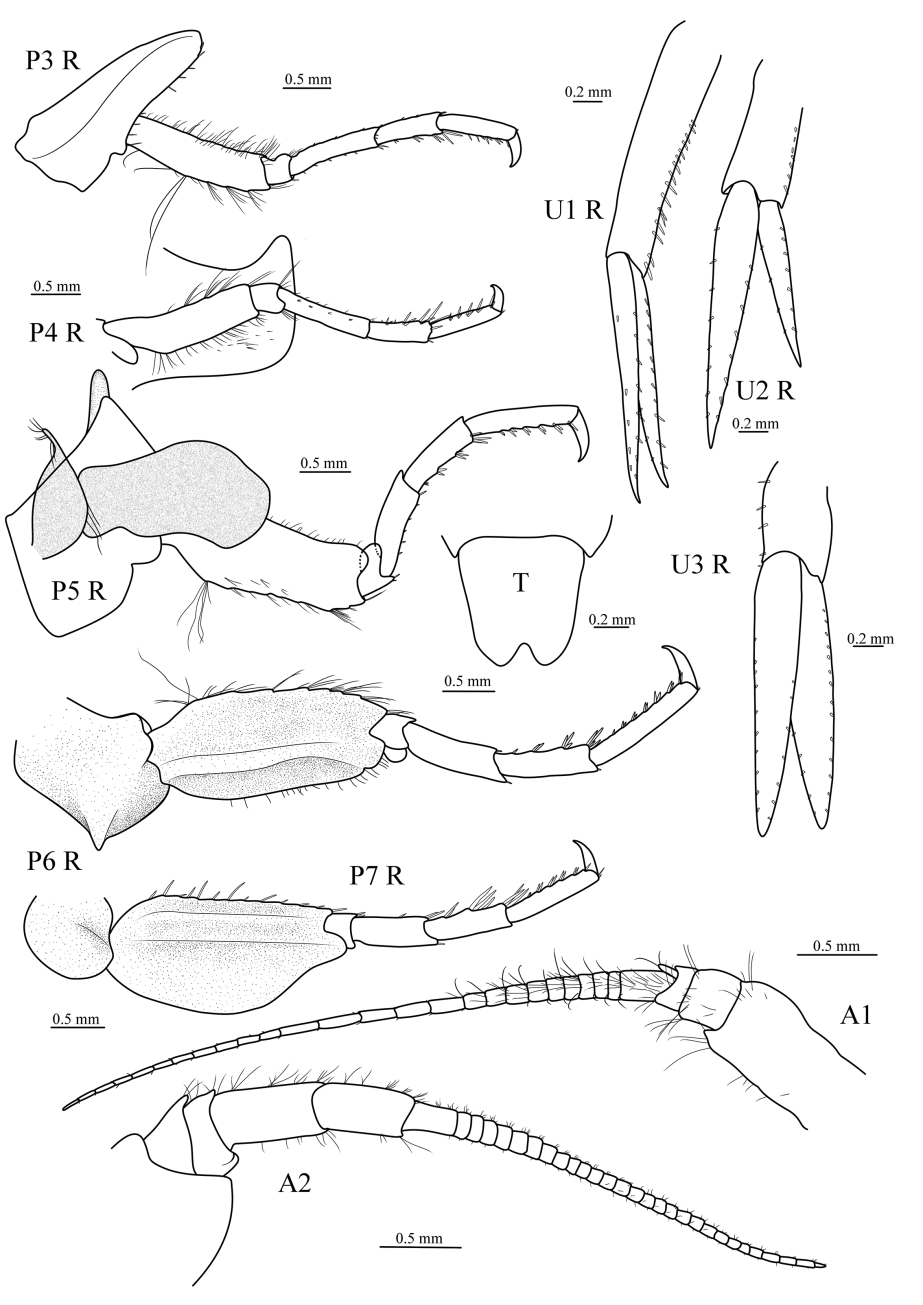
*Epimeria liui* sp. nov., female holotype (17.8 mm) (MBM 286613), P3 R, right pereopod 3; P4 R, right pereopod 4; P5 R, right pereopod 5; P6 R, right pereopod 6; P7 R, right pereopod 7; A1, antenna 1; A2, antenna 2; U1 R, right uropod 1; U2 R, right uropod 2; U3 R, right uropod 3; T, telson.

***Uropods and telson.*** Uropod 1 peduncle subequal in length to rami, outer margin setose; rami subequal in length, margins bearing small robust setae. Uropod 2 peduncle subequal to outer ramus, outer margin setose; outer ramus shorter than inner ramus, both rami outer and inner margins setose. Uropod 3 peduncle much shorter than rami, inner margin with robust setae; rami subequal in length, inner and outer margins of both rami bearing short robust setae. Telson nearly as long as wide, posterior margin notched medially.

**Coloration.** Freshly captured specimen of *Epimeria liui* sp. nov. show distinct orange eyes and rose- to ivory-colored body.

**Etymology.** The species is named in honor of the late Prof. Dr. Ruiyu Liu (J.Y. Liu), the Institute of Oceanology, Chinese Academy of Sciences, for his great contribution to the carcinology of China.

**Distribution.** NW Pacific, unnamed seamount on Caroline Plate at a depth of 813–1242 m.

**Remarks.** Eight *Epimeria* species have been reported from the northern Pacific, including *E. abyssalis* Shimomura & Tomikawa, 2016, *E. cora* J.L. Barnard, 1971, *E. morronei*Winfield et al., 2012, *E. ortizi* Varela & García-Gómez, 2015, *E. pacifica* Gurjanova, 1955, *E. pelagica* Birstein & Vinogradov, 1958, *E. subcarinata* Nagata, 1963 and *E. yaquinae* McCain, 1971. *Epimeria liui* sp. nov. can be distinguished from above species by the following characters: rostrum hardly reaching to the distal margin of first peduncular article of antenna 1; the presence of pyriform pigmented eyes; the projection of coxa 5 not reaching to epimeral plate 1. Actually, *E. liui* sp. nov. more closely resembles *E. bruuni* Barnard, 1961 and *E. horsti* Lörz, 2008, which occur in the southern Pacific, by the produced mid-dorsal carinae starting from pereonite 5 or 6 and having the process on coxa 5 not extending to pleonite 1. The new species differs from *E. bruuni* by the mid-dorsal teeth starting on pereonite 6 and the pleonite 3 not having a large acute mid-dorsal tooth. *Epimeria liui* sp. nov. is especially similar to *E. horsti* for the coloration of the animal body. But it morphologically differs from *E. horsti* by the rostrum not extending to the distal margin of first peduncular article of antenna 1, the anterior cephalic margin having a semicircular lobe, the coxa 5 having a ridge whereas this part in *E. horsti* appears to be smooth (Lörz 2008, figs 1, 5), the mid-dorsal blunt tooth of pereonite 6 not forming a triangular acute tooth as in *E. horsti* (Lörz 2008, fig. 1), the pleonite 3 not having a mid-dorsal tooth, the posteroventral angle of the epimeron 3 not being produced, and by the telson being notched medially. The key to the species of *Epimeria* based on Lörz and Coleman (2014) and Shimomura and Tomikawa (2016) is presented below.

**Figure 4.**
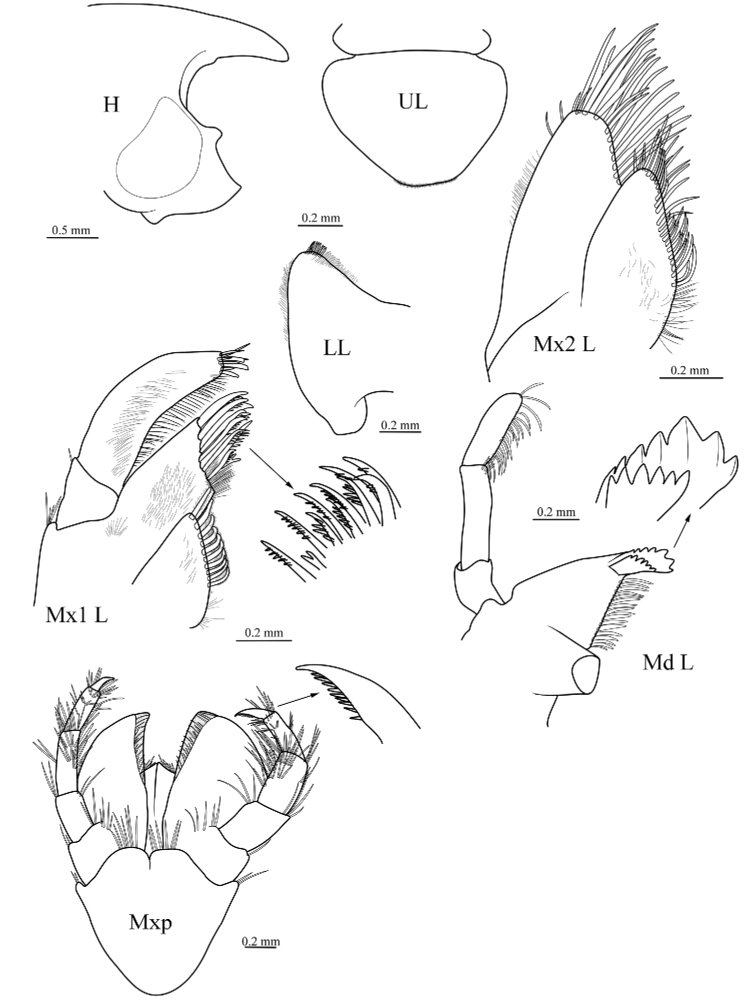
*Epimeria liui* sp. nov., female holotype (17.8 mm) (MBM 286613), H, head; UL, upper lip; LL, lower lip; Md L, left mandible; Mx1 L, left maxilla 1; Mx2 L, left maxilla 2; Mxp, maxilliped.

### Key to the Pacific species of *Epimeria*

**Table:** 

1	Pereon segments lacking dorsal carinae	**2**
–	Pereon segments bearing dorsal carinae	**11**
2	Eyes present	**3**
–	Eyes absent	**8**
3	Urosomite 1 bearing dorsally pointed tooth; rostrum extending beyond first peduncle article of antenna 1	**4**
–	Urosomite 1 lacking dorsally pointed tooth; rostrum not extending beyond first peduncle article of antenna 1	**6**
4	Coxa 5 projection not reaching to epimeral plate 1	***E. cora* J.L. Barnard, 1971**
–	Coxa 5 projection reaching to epimeral plate 1	**5**
5	Head ventral lobe not produced	***E. ortizi* Varela & García-Gómez, 2015**
–	Head ventral lobe produced	***E. pacifica* Gurjanova, 1955**
6	Coxa 5 with protrusion reaching posterior margin of epimeral plate 2; telson not cleft	***E. norfanzi* Lörz, 2011**
–	Coxa 5 not produced; telson cleft	**7**
7	Telson with deep and broad V-shaped excavation	***E. pelagica* Birstein & M. Vinogradov, 1958**
–	Telson with deep and narrow Y-shaped excavation	***E. abyssalis* Shimomura & Tomikawa, 2016**
8	Coxa 5 produced	**9**
–	Coxa 5 not produced	***E. yaquinae* McCain, 1971**
9	Pleonites 1–3 with dorsal carinae; pleonite 3 not dorsally produced; coxae 1–3 ventrally rounded	**10**
–	Pleonites 1–2 smooth; pleonite 3 dorsally produced; coxae 1–3 ventrally pointed	***E. subcarinata* Nagata, 1963**
10	Rostrum not extending beyond first peduncle article of antenna 1	***E. glaucosa* J.L. Barnard, 1961**
–	Rostrum extending beyond second peduncle article of antenna 1	***E. morronei* Winfield, Ortiz & Hendrickx, 2012**
11	Coxa 5 produced	**12**
–	Coxa 5 not produced	**15**
12	Dorsal carinae starting on pereon 4; epimeral plates bearing produced postero-lateral corners and at least two produced lateral teeth each	***E. victoria* Hurley, 1957**
–	Dorsal carinae starting on pereon 6; posterolateral corners of epimeral plates 1 and 2 rounded or weakly produced	**13**
13	Pereonites 6 and 7 laterally smooth; coxa 1 ventrally rounded	**14**
–	Pereonites 6 and 7 laterally bearing projections; coxa 1 ventrally subquadrate	***E. emma* Lörz & Coleman, 2014**
14	Rostrum not extending to distal margin of first peduncular article of antenna 1	***E. liui* sp. nov.**
–	Rostrum beyond distal margin of first peduncular article of antenna 1	***E. horsti* Lörz & Coleman, 2014**
15	Double dorsal carinae present on pleonites 1–3	**16**
–	Single dorsal carinae present on pleonites 1–3	***E. bruuni* J.L. Barnard, 1961**
16	Pleonites laterally smooth	***E. sophie* Lörz & Coleman, 2014**
–	Pleonites laterally bearing several projections	**E. (Metepimeria) ashleyi (Lörz, 2012)**

## Supplementary Material

XML Treatment for Epimeria

XML Treatment for Epimeria liui
